# Competence and Training Needs in Infectious Disease Emergency Response Among Chinese Nurses: Cross-Sectional Study

**DOI:** 10.2196/62887

**Published:** 2024-11-18

**Authors:** Dandan Zhang, Yong-Jun Chen, Tianxin Cui, Jianzhong Zhang, Si-Ying Chen, Yin-Ping Zhang

**Affiliations:** 1 Affiliated Nanhua Hospital, Hengyang Medical School, University of South China, Hengyang, China; 2 School of Nursing, Xi'an Jiaotong University Health Science Center, No.76, Yanta West Road, Xi'an, Shaanxi, 710061, China, 86 29 8265 7015, 86 29 8265 7015; 3 Nursing Studies, School of Health in Social Science, University of Edinburgh, Edinburgh, United Kingdom; 4 Department of Scientific Education, The First People's Hospital of Datong, Datong, China

**Keywords:** competence, preparedness, infectious disease emergency, Chinese, nurse, cross-sectional study, COVID-19, pandemic, public health, health crises, emergency response, emergency preparedness, medical institution, health care worker, linear regression

## Abstract

**Background:**

In recent years, the frequent outbreaks of infectious diseases and insufficient emergency response capabilities, particularly issues exposed during the COVID-19 pandemic, have underscored the critical role of nurses in addressing public health crises. It is currently necessary to investigate the emergency preparedness of nursing personnel following the COVID-19 pandemic completely liberalized, aiming to identify weaknesses and optimize response strategies.

**Objective:**

This study aimed to assess the emergency response competence of nurses, identify their specific training needs, and explore the various elements that impact their emergency response competence.

**Methods:**

Using a multistage stratified sampling method, 5 provinces from different geographical locations nationwide were initially randomly selected using random number tables. Subsequently, within each province, 2 tertiary hospitals, 4 secondary hospitals, and 10 primary hospitals were randomly selected for the survey. The random selection and stratification of the hospitals took into account various aspects such as geographical locations, different levels, scale, and number of nurses. This study involved 80 hospitals (including 10 tertiary hospitals, 20 secondary hospitals, and 50 primary hospitals), where nurses from different departments, specialties, and age groups anonymously completed a questionnaire on infectious disease emergency response capabilities.

**Results:**

This study involved 2055 participants representing various health care institutions. The nurses’ mean score in infectious disease emergency response competence was 141.75 (SD 20.09), indicating a moderate to above-average level. Nearly one-fifth (n=397, 19.32%) of nurses have experience in responding to infectious disease emergencies; however, they acknowledge a lack of insufficient drills (n=615,29.93%) and training (n=502,24.43%). Notably, 1874 (91.19%) nurses expressed a willingness to undergo further training. Multiple linear regression analysis indicated that significant factors affecting infectious disease emergency response competence included the highest degree, frequency of drills and training, and the willingness to undertake further training (*B*=−11.455, 7.344, 11.639, 14.432, 10.255, 7.364, and −11.216; all *P*<.05). Notably, a higher frequency of participation in drills and training sessions correlated with better outcomes (*P*<.001 or *P*<.05). Nurses holding a master degree or higher demonstrated significantly lower competence scores in responding to infectious diseases compared with nurses with a diploma or associate degree (*P*=.001). Approximately 1644 (80%) of the nurses preferred training lasting from 3 days to 1 week, with scenario simulations and emergency drills considered the most popular training methods.

**Conclusions:**

These findings highlight the potential and need for nurses with infectious disease emergency response competence. Frequent drills and training will significantly enhance response competence; however, a lack of practical experience in higher education may have a negative impact on emergency performance. The study emphasizes the critical need for personalized training to boost nurses’ abilities, especially through short-term, intensive methods and simulation drills. Further training and tailored plans are essential to improve nurses’ overall proficiency and ensure effective responses to infectious disease emergencies.

## Introduction

Nurses have become the backbone in responding to infectious disease outbreaks on a global scale. They play an indispensable role in pandemic control, isolation, containment, infection prevention, and public health protection [1], as evidenced by recent significant outbreaks such as COVID-19, SARS, and Ebola. Nurses must possess the skills to handle emergencies, including rapid response, effective communication, teamwork, and decision-making [2-4]. As frontline fighters against the pandemic, their ability to manage and respond to infectious disease emergencies is crucial for ensuring the safety of both patients and themselves [5].

Nurses’ response to infectious disease outbreaks is crucial, so the study of their emergency response capabilities has received significant attention. Previous studies indicate deficiencies in nurses’ preparedness and response to public health emergencies, including a lack of emergency knowledge and inadequate crisis awareness [6,7]. Core competencies in disaster preparedness among nursing personnel and staffing shortages are deemed primary factors influencing the effectiveness of disaster response [8-10]. Factors such as hospital grade, educational background, professional title, sex, work experience, and relevant training may contribute to differences in nurses’ emergency response capabilities during disasters or public health emergencies. At the same time, their emergency response capabilities are also influenced by their level of emergency knowledge and attitude [8,10-12]. However, there is a considerable disparity in the emergency response capabilities of nurses when facing emerging infectious disease outbreaks. In China, secondary and tertiary general hospital nurses demonstrate moderate to below-average emergency response capabilities during major infectious disease outbreaks [11-14]. In contrast, their counterparts in primary health care institutions exhibit even lower proficiency levels [15,16]. Furthermore, differences in emergency response capabilities are observed among nurses in specialized infectious disease hospitals and Traditional Chinese Medicine hospitals [12,17]. These discrepancies include insufficient professional training and drills, lack of emergency supplies, and inadequate emergency support systems [2,10,18].

Research indicated that insufficient professional knowledge correlates with heightened anxiety and stress levels among nurses during the pandemic [19,20]. Moreover, these psychological issues can affect their decision-making and response abilities [21,22]. Past experiences with outbreaks of infectious diseases like SARS, H7N9, and Middle East respiratory syndrome have shown that a shortage of experienced health care professionals is a significant obstacle in effectively responding to sudden infectious disease events [23,24]. The COVID-19 pandemic has revealed deficiencies in many countries’ responses, particularly in the emergency response capabilities of nursing staff. The early investigation of the COVID-19 outbreak revealed that most nurses involved in support lacked experience and preparation, had insufficient emergency response capability, faced prominent psychological issues, lacked knowledge of ethics and laws, and had limited exposure to scenario-based training [15,20-22,25,26]. These issues may impair nurses’ ability to effectively respond to infectious disease crises, necessitating great attention from relevant authorities. Enhancing nurses’ emergency response capabilities through ongoing training, improving resource readiness, and establishing robust emergency plans are crucial measures to comprehensively enhance nursing emergency response preparedness and address this issue effectively.

Enhancing nursing emergency response capabilities is crucial in improving the efficiency and quality of health care services during infectious disease outbreaks, alleviating societal panic, and maintaining public health and social stability [27]. Throughout the COVID-19 pandemic, medical institutions in China faced immense pressure and challenges. China’s containment strategies advanced from initial precision-based prevention measures to a phase of normalized control, culminating in a complete easing of restrictions in 2023. Hospitals have increased related training efforts considering nurses’ weaknesses in infection control. Therefore, following the easing of pandemic restrictions, assessing the competency of nurses in emergency response and identifying their training requirements can offer valuable insights to nursing managers and decision-makers. This assessment helps identify shortcomings and obstacles faced by nurses during infectious disease emergencies and explores various factors affecting their emergency capabilities, contributing to the optimization of management strategies and enhancement of nursing efficacy. Evaluating nurses’ emergency response competence after the relaxation of COVID-19 restrictions holds significant importance in improving existing nursing protocols [11]. Early identification of problems followed by targeted intervention and training can significantly improve the emergency preparedness of nurses, preparing them well for potential future infectious disease outbreaks. Thus, it is urgent and necessary to investigate nurses’ emergency response competence and training needs.

This study aimed to assess the emergency response competence of nurses during the COVID-19 pandemic completely liberalized, identify their specific training needs, and explore the various elements that impact their emergency response competence. The results will serve as a foundation for developing future training programs.

## Methods

### Study Design

The study used a cross-sectional survey to investigate nurses working in public hospitals located in 5 provinces across different regions of China: Hunan in the central region, Guangdong in the southern region, Sichuan in the western region, Shanxi in the northern region, and Zhejiang in the eastern region. Data collection occurred from January to March 2024, focusing on assessing nurses’ infectious disease emergency response competency and obtaining their training needs.

Chinese hospitals are divided into 3 levels: primary, secondary, and tertiary. Primary hospitals provide community medical services, generally have 10‐99 beds and at least 5‐40 nurses, and are usually located in communities or rural areas. Secondary hospitals offer regional medical services in multiple communities, typically have 100‐499 beds and 40‐200 or more nurses, and are usually situated in towns. Tertiary hospitals are comprehensive medical centers that offer services across regions, provinces, cities, or even nationally, with over 500 beds and employing 400‐2000 or more nurses. They are commonly located in urban areas. To meet the sample size requirements of the study, this study plans to include tertiary hospitals with 800‐1000 or more nurses, secondary hospitals with 300‐400 or more nurses, and primary hospitals with 10‐20 or more nurses for investigation.

### Sample and Setting

The inclusion criteria were as follows: (1) registered nurse (participants must possess a valid nursing license), (2) nurses with over 1 year of clinical experience, and (3) all participants are voluntary and must sign an informed consent form before participating.

The exclusion criteria were as follows: (1) absence of a nursing license, (2) noncooperation with the investigation, (3) more than 5% missing values in submitted questionnaires will be excluded from the analysis, and (4) completion of the questionnaire in less than 2 minutes will be deemed ineligible for analysis.

The required sample size is calculated using the following formulae:


(1) $$n_{1}={\left(\frac{Z_{\alpha /2}\sigma }{\delta }\right)}^{\text{2}}$$

(2) $$n_{2}=n_{1}N/\left(N+n_{1}\right)$$

With Z_α/2_=1.96, σ=20.91 [28], *δ*=1, formula 1 yields an initial sample size (n1) of 1680. When formula 2 was applied with a population size (N) of 20,000, the sample size (n2) was determined to be 1550. The sample size is increased by 10% to 20% to account for potential errors, giving a final range of 1705 to 1859. Adopting the upper limit of this range ensures a sufficient sample size; consequently, the study should include no fewer than 1859 samples.

This study used a multistage stratified sampling approach. Initially, China was divided into 5 geographical regions as follows: central, southern, western, northern, and eastern. One province was randomly chosen from each region using random number tables, resulting in the selection of Hunan (central), Guangdong (southern), Sichuan (western), Shanxi (northern), and Zhejiang (eastern). In the second phase, based on the hospital levels, sizes, bed capacities, and nurse numbers specified by the National Health Commission, we selected all tertiary hospitals with 800 to 1000 nurses or more, secondary hospitals with 300 to 400 nurses or more, and primary hospitals with 10 nurses or more in these 5 provinces. Subsequently, hospitals of different levels were randomly selected from these 5 provinces, ensuring representation from each category. This included 2 tertiary hospitals per province, 4 secondary hospitals per province, and 10 primary hospitals per province. This comprehensive selection process yielded a total sample of 80 hospitals, which included 10 tertiary hospitals, 20 secondary hospitals, and 50 primary hospitals. The total pool of approximately 20,000 nurses represented a wide range of age groups, specialties, experience levels, and departments within public hospitals, ensuring a diverse and representative sample for the study.

During the data collection phase, heads of nursing departments in each hospital distributed online questionnaires via the hospital’s WeChat (Tencent) group for nurses, allowing voluntary online participation. A total of 2213 nurses completed the survey online. After excluding invalid responses, 2055 valid questionnaires were analyzed.

### Questionnaire and Study Variables

The survey in this study used the health care workers’ infectious disease emergency response competence questionnaire developed by Kan [29] in 2018. This questionnaire was developed based on an extensive literature review and expert input in public health emergencies and disaster relief nursing [27,29,30]. The final version was established after pilot testing and improvements with 328 nurses. Research by Song et al [11,17,31,32] used this questionnaire to survey nurses in various medical institutions, confirming its practical application in different environments. These studies’ findings have demonstrated that the questionnaire is reliable and valid and has been successfully applied in relevant studies in China.

The questionnaire comprises 3 main sections as follows.

Part 1 focuses on self-assessment of infectious disease emergency response competence, including 3 primary, 11 secondary, and 36 tertiary indicators. The specific indicators and their scoring details are as follows: The primary indicators include prevention competence, preparedness competence, and response competence. Prevention competence mainly measures knowledge of basic prevention and control of infectious diseases, with 3 tertiary indicators under it. Preparedness competence includes 2 secondary indicators related to emergency plans and laws and regulations, with 4 tertiary indicators under it. Response competence includes 6 secondary indicators: monitoring, reporting, medical response, public health response, risk communication, and response to sudden incidents under specific conditions, with 29 tertiary indicators. Each item is scored at a Likert level 5, ranging from 1 to 5 points for “completely unfamiliar” to “very familiar,” with a total score of 36‐180. The higher the score, the better the level of emergency response capability. Scores below 60% (36‐108 points) indicated low level, scores between 60% and 79% (108‐143 points) indicated midlevel, and scores above 80% (144‐180 points) were considered high level [17,33,34]. The questionnaire demonstrates good overall content validity, with a validity rate of 0.872. The internal consistency reliability coefficients for each dimension range from 0.757 to 0.957, and the test-retest reliability ranges from 0.448 to 0.772 [29]. In this study, the Cronbach α value was 0.93. According to expert evaluation, the content validity index was 0.87 [8,11,30]. This study provides strong evidence of the questionnaire’s reliability and validity.

Part 2 focuses on infectious disease emergency experience and training requirements. This part includes 7 multiple-choice questions covering topics such as experience in responding to infectious disease outbreaks and other crises (such as earthquakes, fires, etc), training experience, and training requirements (including willingness to undertake further training, acceptable training duration, and preferred training methods).

Part 3 is demographic characteristics. It mainly includes sex, age, department of work, hospital grade, position, professional title, highest educational degree, work experience, etc.

### Data Collection

The questionnaire was published on Sojump, the most widely used survey platform in China, and it generated an electronic report (e-poster) containing a QR code and survey link. This e-poster and link were sent to the nursing department director at the selected hospital, who then shared them with the hospital’s nursing staff through a WeChat group to facilitate recruitment. Nurses could scan the QR code or click the link on their smartphones to access the survey, including an explanatory letter and a request for informed consent. At this stage, they were assured of anonymity. Nurses could proceed with the study after selecting the “I am informed and agree to participate” option. The system implemented measures to prevent repetitive responses by permitting submission from each unique mobile number only once and mandating completion of all survey items before submission. The research team conducted data quality control after the survey was completed.

### Statistical Analysis

Data were entered into SPSS (version 25.0; IBM Corp) for statistical analysis. Demographic data were characterized using descriptive statistics. For continuous variables, if the data followed a normal distribution, it was represented by the mean and SD. For nonnormally distributed data, the median and IQR were used for description. Categorical variables were represented as percentages. Group differences were assessed using a 2-tailed Student *t* test and ANOVA. A multiple linear regression analysis was conducted to examine variables influencing infectious disease emergency response capabilities, with the total score designated as the dependent variable and significant factors from the one-way ANOVA (eg, demographic characteristics, emergency response experience, and training experience) serving as independent variables. *P* values less than .05 (2-tailed) were deemed to indicate statistical significance.

### Ethical Considerations

The study adhered to the Declaration of Helsinki standards and received ethics approval from the ethics committee of Affiliated Nanhua Hospital, University of South China (approval number 2024-KY-075). Informed consent was obtained online, and participation was voluntary, with assurance of anonymity and confidentiality in data handling. Participants had the right to withdraw at any time, and access to data was restricted to the lead researcher.

This study was a cross-sectional survey focused on assessing infectious disease emergency response competence among nurses and did not involve patients.

## Results

### Demographic Data

In this study, 2055 nurses were included, with the vast majority being female (n=1925, 93.67%) and males totaling 130 (6.33%). Of these nurses, 68.86% (n=1415) worked in tertiary hospitals. Approximately 1014 (49.34%) of the nurses had less than 5 years of work experience, 1126 (54.79%) held primary professional titles, and 1256 (61.12%) possessed a bachelor’s degree or higher. Univariate analysis revealed significant associations between age, highest level of education, and competence scores in responding to infectious disease emergencies (all *P*<.01). The details are shown in Table 1.

**Table 1.** The demographic characteristics and competence scores of the nurses who participated in an observational cross-sectional study on responding to infectious disease emergencies. This study was conducted across 5 provinces in China from January to March 2024 (N=2055). The data in this table can be considered approximately normally distributed, as all variables have absolute kurtosis values of less than 10 and absolute skewness values of less than 3.

Variables	Total (N=2055), n (%)	Score, mean (SD)	*F* test (*df*)/*t* test (*df*)	*P* value
Sex			−1.585 (2053)	.11
Male	130 (6.33)	137.97 (35.28)		
Female	1925 (93.67)	142.00 (27.53)		
Age (years)			4.453 (3, 2051)	.004
≤25	779 (37.90)	139.32 (29.25)		
26‐30	286 (13.92)	145.67 (27.80)		
31‐40	704 (34.26)	143.13 (27.50)		
≥41	286 (13.92)	141.03 (25.98)		
Clinical experience (years)			2.449 (3, 2051)	.06
≤5	1014 (49.34)	140.20 (29.21)		
6‐10	406 (19.76)	144.11 (27.58)		
11‐20	494 (24.04)	143.19 (26.39)		
≥21	141 (6.86)	141.02 (26.59)		
Highest degree			6.924 (2, 2052)	.001
Diploma or associate degree	799 (38.88)	140.93 (29.34)		
Bachelor degree	1186 (57.71)	142.95 (26.84)		
Master and doctor degree	70 (3.41)	130.66 (31.60)		
Professional title			0.200 (2, 2052)	.82
Primary	1126 (54.79)	142.09 (29.495)		
Intermediate	767 (37.32)	141.26 (28.87)		
Senior	162 (7.88)	141.67 (26.30)		
Position			0.281 (2, 2052)	.76
Nurse	1854 (90.22)	141.90 (28.18)		
Head nurse	167 (8.13)	140.25 (26.59)		
Director of the nursing department	34 (1.65)	140.85 (30.69)		
Health care system			0.351 (2, 2052)	.70
Primary hospitals	249 (12.12)	140.61 (28.27)		
Secondary hospitals	391 (19.03)	141.29 (29.23)		
Tertiary hospitals	1415 (68.86)	142.07 (27.74)		
Hospital department of work			1.837 (5, 2049)	.10
Medical ward	494 (24.04)	144.47 (29.33)		
Surgical ward	471 (22.92)	143.05 (26.32)		
Obstetric and gynecological department	316 (15.38)	139.54 (25.80)		
Pediatrics department	252 (12.26)	142.33 (27.68)		
Emergency and critical care unit	164 (7.98)	143.07 (25.72)		
Others^a^	358 (17.42)	140.15 (29.30)		

a Traditional Chinese Medicine Department, operating room, outpatient services, etc.

### Nurses’ Competence in Responding to Infectious Disease Emergencies

The mean total score for the infectious disease emergency response competence of nurses was 141.75 (SD 20.09) points, with 78.8% of the scores indicating that their performance was moderate and close to a high level. The score for the dimension of preventive competence was 12.08 (SD 2.41), with 80.5% of the scores showing a relatively high level of capability. The scores for preparedness competence and response competence were 15.37 (SD 3.56) and 114.30 (SD 22.77), respectively, falling within the 60%‐79% score range, indicating a moderate level of proficiency. Table 2 contains more results.

**Table 2.** The scores from a cross-sectional survey assessing the competence of 2055 nurses from 5 Chinese provinces in responding to infectious disease emergencies. The survey was conducted between January and March. The data in this table can be considered approximately normally distributed, as all variables have absolute kurtosis values of less than 10 and absolute skewness values of less than 3.

Variable	Score range	Score, mean (SD)	Kurtosis	Skewness
Prevention competence	3‐15	12.08 (2.41)	−0.772	1.108
Preparedness competence	4‐20	15.37 (3.56)	−0.517	−0.029
Emergency plans and	2‐10	7.83 (1.79)	−0.633	0.127
Laws and regulations	2‐10	7.54 (1.88)	−0.467	−0.173
Response competence	29‐145	114.30 (22.77)	−0.509	0.357
Monitoring	3‐15	11.39 (2.70)	−0.433	−0.088
Reporting	4‐20	15.34 (3.55)	−0.46	−0.096
Medical response	6‐30	23.41 (5.02)	−0.466	0.08
Public health response	12‐60	49.36 (9.23)	−0.884	1.263
Risk communication	1‐5	3.87 (0.93)	−0.553	−0.086
Response to sudden incidents under specific conditions	4‐20	10.94 (3.06)	−0.326	−0.664
Total	36‐180	141.75 (20.09)	−0.504	0.39

### Highest and Lowest Scoring Aspects of Nurses’ Competence in Responding to Infectious Disease Emergencies

The item achieving the highest score was “Implementation of Hand Hygiene,” followed by “Management of Medical Waste” and “Proper Use of Personal Protective Equipment,” among others. In contrast, “Critical Precautions for International Rescue Operations” received the lowest rating, followed by “Response to Biological Terrorist Threats” and “Recognition of Symptoms and Diagnosis of Infectious Diseases,” among other items. Table 3 contains more results.

**Table 3.** Presents the highest- and lowest-scoring aspects of nurses’ competence in responding to infectious disease emergencies. This cross-sectional survey assessed the competence in responding to infectious disease emergencies of 2055 nurses from 5 Chinese provinces between January and March. The data in this table can be considered approximately normally distributed, as all variables have absolute kurtosis values of less than 10 and absolute skewness values of less than 3.

Variables	Score range	Score, mean (SD)	Kurtosis	Skewness
Top 5 high-scoring items				
Item 26: implementation of hand hygiene	1‐5	4.33 (0.80)	−1.258	2.017
Item 27: management of medical waste	1‐5	4.29 (0.80)	−1.181	1.832
Item 23: donning and doffing personal protective equipment	1‐5	4.19 (0.83)	−0.998	1.23
Item 24: procedures for exposure to patient blood and body fluids	1‐5	4.17 (0.83)	−0.94	1.012
Item 29: methods for environmental disinfection	1‐5	4.16 (0.83)	−0.87	0.832
Bottom 5 low-scoring items				
Item 35: response to infectious disease outbreaks after natural disasters	1‐5	3.75 (0.98)	−0.431	−0.479
Item 12: fill out the infectious disease report card	1‐5	3.74 (1.01)	−0.44	−0.384
Item 9: identification of infectious disease symptoms and determination of diseases	1‐5	3.72 (0.98)	−0.442	−0.311
Item 34: response to biological terrorist attacks	1‐5	3.62 (1.07)	−0.403	−0.609
Item 36: understanding aspects of participating in international rescue operations	1‐5	3.56 (1.13)	−0.401	−0.695

### Nurses’ Experiences in Emergency Response and Their Training Needs

Among the respondents, 397 (19.32%) had experience in responding to infectious diseases, scoring significantly higher (mean 146.00, SD 27.00) than those without such experience (mean 140.70, SD 28.26; *P*=.001). Conversely, experience in handling other crises such as earthquakes and fires was present in 257 (12.51%) of nurses, with no significant difference in scores compared with those without experience (*P*=.10). The majority of nurses had participated in infectious disease emergency drills (1440/2055, 70.07%) and training (1553/2055, 75.57%) within the past 5 years, with over 40% attending 1‐4 emergency drills and training sessions, while more than 20% had participated in 5 or more training and drill sessions. Nevertheless, 615 (29.93%) had not engaged in drills, and 502 (24.43%) had not undergone formal training during this period. The frequency of emergency drills significantly impacted scores (*P*<.001), with higher frequencies correlating with better outcomes. Similarly, the frequency of infectious disease training also showed significant differences in scores (*P*<.001), with more sessions linked to higher scores. Furthermore, nurses willing to undertake further training scored significantly higher (mean 142.74, SD 27.53) than those uncertain (mean 126.64, SD 28.28) or unwilling (*P*<.001). Table 4 contains more results.

Table 5 shows the specific training needs of nurses in responding to infectious disease emergencies. A total of 1644 (80%) nurses preferred training programs that last no longer than a week, with 916 (44.57%) favoring sessions that span just 3 days. When it came to training methodologies, they could opt for multiple choices. Scenario-based simulation training emerged as the favorite, with 1481 (72.07%) nurses selecting this option, and it was closely followed by emergency drills, with 1410 (68.61%) nurses selecting this option. Theoretical lectures and skill training also rank highly, enjoyed by 1386 (67.45%) and 1313 (63.89%) of nurses, respectively.

**Table 4.** Emergency response experience and training requirements among nurses in 5 provinces of China (January-March 2024, N=2055). The data in this table can be considered approximately normally distributed, as all variables have absolute kurtosis values of less than 10 and absolute skewness values of less than 3.

Variables	Total, n (%)	Score, mean (SD)	*F* test (*df*)/*t* test (*df*)	*P* value
Experience in emergency response to infectious diseases			3.333 (2053)	.001
Yes	397 (19.32)	146.00 (27.00)		
No	1658 (80.68)	140.70 (28.26)		
Experience in emergency response to other crises (earthquakes, fires, and other disasters or accidents)			1.634 (2053)	.10
Yes	257 (12.51)	144.40 (30.06)		
No	1798 (87.49)	141.40 (27.78)		
Emergency drills for infectious disease in the last 5 years (frequency)			44.974 (3, 2051)	<.001
0	615 (29.93)	132.91 (27.69)		
1‐4	895 (43.55)	141.87 (27.35)		
5‐9	390 (18.98)	151.40 (26.22)		
≥10	155 (7.54)	151.81 (27.10)		
Training for infectious disease in the last 5 years (frequency)			43.259 (3, 2051)	<.001
0	502 (24.43)	132.58 (28.47)		
1‐4	831 (40.44)	139.94 (27.61)		
5‐9	469 (22.82)	150.45 (25.32)		
≥10	253 (12.31)	149.72 (27.08)		
Willingness to undertake further training			18.4 (2, 2052)	<.001
Yes	1874 (91.19)	142.74 (27.53)		
Uncertain	116 (5.65)	126.64 (28.28)		
No	65 (3.16)	140.00 (35.31)		

**Table 5.** Illustrates the nurses’ perspectives on training duration and preferred methods based on a survey conducted across 5 provinces in China from January to March 2024 (N=2055).

Variables		Values, n (%)
How long do you think training on responding to infectious disease emergencies should last for optimal effectiveness?		
	1‐3 days	916 (44.57)
	4 days-1 week	728 (35.43)
	1 week-1 month	243 (11.82)
	>1 month	168 (8.18)
The training methods in which you prefer to participate (multiple choices)		
	Scenario-based simulation training	1481 (72.07)
	Emergency drills	1410 (68.61)
	Theoretical lectures	1386 (67.45)
	Skill training	1313 (63.89)
	Role-playing training	978 (47.59)
	e-Learning	961 (46.76)
	Virtual reality simulation training	828 (40.29)
	Quiz and preemptive answer competition	560 (27.25)
	Desktop maneuvers	536 (26.08)
	Self-study	409 (19.90)
	Others	25 (1.22)

### Multiple Linear Regression Analysis of Factors Affecting Nurses’ Emergency Response Competence for Infectious Diseases

Table 6 presents the results of a multiple linear regression analysis on factors influencing nurses’ emergency infectious disease preparedness. The regression analysis used the total score of nurses’ competence in handling infectious disease emergencies as the dependent variable. The significant variables identified from Student *t* tests and ANOVA in Tables1  4 were included as independent variables in the multiple linear regression analysis, encompassing the following 6 variables: age (*F*_3,2051_=4.453, *P*=.004), highest education level (*F*_2,2052_=6.924, *P*=.001), experience in infectious disease emergency response (*t*_2053_=3.333, *P*=.001), frequency of infectious disease emergency drills in the past 5 years (*F*_3,2051_=44.974, *P*<.001), frequency of infectious disease training sessions in the past 5 years (*F*_3,2051_=43.259, *P*<.001), and willingness to undertake further training (*F*_2,2052_=18.4, *P*<.001). A correlation analysis was conducted on all independent variables before regression analysis. The correlation coefficients among the independent variables were below 0.7, with all *P* values above .05, indicating weak intervariable correlations and the absence of multicollinearity.

**Table 6.** Multiple linear regression analysis of factors affecting infectious disease emergency response competence among nurses: survey conducted from January to March 2024 in 5 provinces of China (N=2055). Dependent variable: total score of nurses’ competence in infectious disease emergency response. Assigning values to independent variables was as follows: age, entered as original values; highest degree, with diploma or associate degree as the reference, setting 2 dummy variables; participation in infectious disease response, yes=1, no=0; frequency of drills in the past 5 years, with 0 times as the reference, setting 3 dummy variables; frequency of attending infectious disease training in the past 5 years, with 0 times as the reference, setting 3 dummy variables; willingness for further training, with unwilling as the reference, setting 2 dummy variables. Adjusted *R*^2^=0.087, the Durbin-Watson value stands at 1.947. *F*_12,2042_=17.322, *P*<.001. The model is statistically significant overall. The minimum tolerance was 0.369 (all larger than 0.1), and the maximum variance inflation factor (VIF) was 2.879 (all less than 5), indicating that there was no notable multicollinearity problem.

Variables	*B*	*SE*	*β*	*t* test (*df*)	*P* value	Tolerance	VIF
Constant	133.748	5.33	—^a^	25.094	<.001	—	—
Age	−0.138	0.081	−.041	−1.696 (2054)	.09	0.749	1.335
Highest degree							
Bachelor degree	−2.003	1.403	−.035	−1.428 (2052)	.15	0.73	1.371
Master and doctor degree	−11.455	3.385	−.074	−3.384 (2052)	.001	0.93	1.076
Experience in emergency response to infectious	0.599	1.612	.008	0.372 (2053)	.71	0.866	1.155
Emergency drills for infectious disease in the last 5 years (frequency)							
1‐4	7.344	2.101	.13	3.495 (2051)	<.001	0.323	3.097
5‐9	11.639	2.692	.163	4.324 (2051)	<.001	0.315	3.179
≥10	14.432	3.579	.136	4.032 (2051)	<.001	0.392	2.549
Training for infectious disease in the last 5 years (frequency)							
1‐4	1.743	2.251	.03	0.774 (2051)	.44	0.287	3.483
5‐9	10.255	2.73	.153	3.757 (2051)	<.001	0.267	3.745
≥10	7.364	3.334	.086	2.209 (2051)	.03	0.292	3.424
Willingness to undertake further training							
Uncertain	−11.216	4.176	−.092	−2.686 (2052)	.007	0.377	2.65
Yes	3.215	3.396	.032	0.947 (2052)	.34	0.378	2.643

a Not applicable.

The results indicated that compared with nurses holding a diploma/associate degree, those with a master or doctor degree had significantly lower competence scores (*B=*−11.455, *P*=.001). Compared with nurses who did not participate in drills, those who participated in 1‐4 drills in the past 5 years scored 7.344 points higher (*B*=7.344, *P*<.001). Similarly, those who participated in 5‐9 drills scored 11.639 points higher (*B*=11.639, *P*<.001), and nurses who participated in 10 or more drills scored 14.432 points higher (*B*=14.432, *P*<.001). Furthermore, compared with nurses who did not receive any training, those who participated in 5‐9 training sessions in the past 5 years scored 10.255 points higher (*B*=10.255, *P*<.001), and those who completed 10 or more training sessions scored 7.364 points higher (*B*=7.364, *P*=.03). Additionally, in terms of willingness to undergo further training, nurses who expressed uncertainty scored significantly lower in competence (*B*=−11.216, *P*=.007).

## Discussion

### Principal Findings

This cross-sectional study, conducted from January to March 2024 across 5 Chinese provinces, evaluated the competence of nurses in responding to infectious disease emergencies, identified their training needs, and analyzed key determinants affecting their response competence. A total of 2055 nurses participated in the survey and provided valid responses. The study results demonstrate that nurses’ mean score for infectious disease emergency response competence was 141.75 (SD 20.09), slightly above average. This score reflects the situation following the comprehensive lift of COVID-19 restrictions. It is higher than the results reported by studies during the period of precise COVID-19 prevention and control (mean 120.83, SD 11.69 points) [31] and the pre-COVID-19 period (mean 116.13, SD 22.84 points) [30]. Although the nurses’ emergency response competence during these 3 periods ranged from 60% to 79%, indicating a medium level, our study’s score shows an improvement over the previous 2 periods, suggesting potential for further enhancement. Nearly one-fifth (n=397, 19.32%) of nurses have experience in responding to infectious disease emergencies; however, they acknowledge a lack of adequate training (n=502,24.43%) and drills (n=615, 29.93%). The frequency of emergency drills and training significantly impacts performance scores (all *P*<.001 or *P*<.05, Tables4  6). Higher frequencies of such drills and training correlate with better results. Additionally, 1874 (91.19%) of the nurses expressed willingness to receive further training. These findings highlight the potential and necessity for nurses to enhance their response competence in infectious disease emergencies, requiring the urgent implementation of more effective training to improve nurses’ comprehensive response in potential future outbreaks.

Several studies have focused on the performance of nurses in emergency preparedness for infectious disease outbreaks. Li et al’s [34] investigation revealed that Chinese nurses demonstrated a firm grasp of COVID-19 knowledge but lacked practical experience, suggesting the need to enhance nurses’ accumulation of practical experience and emergency training. In a survey conducted by McNeill et al [6] in 2020, it was reported that nurses across the United States lacked adequate professional emergency preparedness capabilities. Wang et al’s [12] study surveyed 652 nurses from specialized infectious disease hospitals, showing that 64.57% of nurses had a moderate level of emergency preparedness, knowledge, and attitudes. Furthermore, Song et al’s [11] research in 2021 indicated that the nursing staff’s core infectious disease emergency capabilities were also moderate. These studies recommend enhancing training and improving nurses’ abilities in responding to infectious disease outbreaks. The findings of this study revealed that nurses’ scores in infectious disease emergency response competence were slightly above the moderate level. In all dimensions, the score for prevention competence was the highest, at a high level. In contrast, preparedness competence and response competence scored a moderate level (Table 2), significantly higher than the results of the survey for health care workers conducted by Kan [29] before the COVID-19 outbreak in 2018. These findings suggest a heightened focus on this aspect during the COVID-19 pandemic, leading to progress in nurses’ response to epidemics and increased prevention capability.

The highest-scoring items were the implementation of hand hygiene, management of medical waste, donning and doffing personal protective equipment, procedures for contact with patient blood and body fluids, and environmental disinfection methods (Table 3). It is evident that the rich practical experience gained by nurses during the COVID-19 outbreak significantly enhanced the scores in these areas. The ongoing epidemic has caused nurses to face more practical challenges, improving their experience and skills. The strict implementation of various preventive measures during the epidemic has also increased nurses’ expertise in protection, disinfection, and other aspects [4,35]. Continuous education and practical training are necessary during nonepidemic periods to maintain and strengthen emergency preparedness for nurses.

However, this study revealed that nurses lack experience in drills and training, with 615 (29.93%) not participating in infectious disease emergency drills in the past 5 years and 502 (24.43%) not receiving any infectious disease training. Over 40% of nurses participate in only 1 or fewer drills or training per year (Table 4). Wang et al [12] found that specialized infectious disease hospitals lack routine training for major infectious disease outbreaks, which may lead to inadequate protection by medical staff, increased transmission risks, decreased work efficiency, and ultimately exacerbate the difficulty of epidemic control and the risk of personnel infections [2,36,37]. Alan et al [10] found that nurses’ preparedness under disaster conditions requires regular training and drills to improve. Nayahangan et al [2] emphasized the urgency of developing and implementing training programs during the pandemic to ensure sufficient resources and personnel are rapidly deployed to the front line. Shi et al’s [13] study shows that nurses with advanced training and experience dealing with COVID-19 patients are more willing to care for infected patients. As guidelines continue to update and ward scenarios become increasingly complex, emerging infectious diseases pose more significant challenges for nursing teams. The annual influx of new nurses further highlights the urgency of enhancing training. The results of this study indicate that the higher the frequency of participation in drills and training courses, the better the outcomes (*P*<.05, Table 6). A total of 1874 (91.19%) of nurses in this study are willing to undergo further training to acquire more comprehensive and practical knowledge to improve their preparedness and response competence in dealing with the pandemic, showing their recognition of the importance of ongoing professional development in this critical field. Therefore, to effectively mitigate the impact of emerging infectious diseases, it is imperative to implement ongoing and advanced training programs to enhance health care workers’ knowledge and skills. This is crucial for reducing mortality rates during epidemics and preventing nosocomial infections. Additionally, our study results underscore the need for nurses to enhance their abilities in identifying symptoms, reporting epidemic situations, and handling sudden outbreaks of infectious diseases in specific scenarios such as bioterrorist attacks, infectious disease outbreaks following natural disasters, and international emergency protocols. (Table 3).

This study revealed that nurses prefer training programs lasting 3 days to a week (Table 5) for concentrated and targeted training, which facilitates in-depth learning and practical application. Nurses also favor scenario-based simulations and emergency drills, highlighting the importance of practical and experiential learning. In particular, those that simulate real-life scenarios enable personnel to gain valuable emergency management experience, identify personal weaknesses, and improve future practices [11,38,39]. Additionally, over 40% of nurses support using emerging technologies such as e-learning and virtual reality simulation for training (Table 5). Some studies have shown the positive effects of online training and virtual reality simulation training on epidemic response capabilities [4,40-45].

Research during the COVID-19 period has indicated that the emergency response of nursing personnel to infectious diseases is influenced by their educational background, participation in infectious disease emergency drills, and prior experience in dealing with infectious diseases [11]. However, after 3 years of the pandemic, our study reveals that experience in infectious disease work is no longer the primary influencing factor. Instead, the frequency of drills, training participation, and willingness to engage in further training have become the key factors (Table 6, all *P*<.05). Regular drills and training significantly enhance the emergency response competence of nursing personnel, although a lack of practical experience in higher education may also negatively impact their emergency performance. This underscores the value of consistent training and drills while highlighting future challenges.

To ensure nurses possess the necessary skills and knowledge to address infectious diseases, it is recommended to strengthen ongoing drills and training programs. Furthermore, future research should explore the specific effects of various training types on emergency response capabilities to identify the most effective strategies. This could involve refining drill content, innovating training methods, and increasing nursing personnel’s engagement in training to enhance overall performance. When designing training programs, it is crucial to consider these influencing factors and provide personalized, targeted training to meet nurses’ specific needs [46,47]. These findings reflect the potential and demand of nurses in training for infectious disease emergency response competence. It is essential to strengthen customized, practical training to enhance nurses’ overall abilities and ensure their prompt and effective response to infectious disease emergencies in the future. Future research can further explore the long-term effects of tailored training programs on nurses’ emergency response competence, providing more information for continuous improvement of medical preparedness.

### Conclusion

This study reveals the significant potential of nurses in infectious disease emergency response and their existing training needs. After COVID-19, the emergency response competence of nurses has generally improved, but there remain issues with insufficient drills and training. Our data show that 1874 (91.19%) of nurses are willing to receive additional training, particularly via short-term, intensive, practical methods and scenario simulation drills, which are widely supported. The study found that educational background, frequency of drills and training, and willingness to undergo training directly impact nurses’ emergency performance, with increased frequency of drills and training directly enhancing their response capabilities. However, it is concerning that nurses with a master degree or higher perform worse in infectious disease emergency response compared with those with a diploma or associate degree, highlighting the negative impact of higher education but lack of practical experience.

The study emphasizes that the key to improving nurses’ emergency response capabilities for infectious diseases lies in personalized and practical training programs. Such training not only strengthens the overall emergency response capabilities of nurses but also enables them to respond quickly and effectively to sudden infectious disease events. To achieve this goal, future research should focus on exploring the impact of customized training plans on nurses’ long-term emergency response capabilities, providing robust guidance for the continuous improvement of medical preparedness.

### Limitations

#### The Sample’s Representativeness is Somewhat Limited

Although this study includes nurses from multiple regions, regional distribution and willingness to participate may result in the specific needs of particular areas or hospitals not being adequately reflected.

#### Potential Biases in Self-Reported Data

The study relies on nurses’ self-assessment of their competence in responding to infectious disease emergencies, which may be influenced by social desirability effects and recall bias, potentially leading to overestimating or underestimating their competencies. However, the health care workers’ infectious disease emergency response competence questionnaire is a validated tool with high internal consistency and reliable testing outcomes. Future research should incorporate more objective measurement methods, such as field tests or simulation exercises, to more accurately evaluate nurses’ capabilities in emergencies related to infectious diseases. Additionally, using comprehensive evaluation approaches would help mitigate the impact of potential biases.
